# Impact of Lactic Acid Bacteria Fermentation on Phenolic Compounds and Antioxidant Activity of Avocado Leaf Extracts

**DOI:** 10.3390/antiox12020298

**Published:** 2023-01-28

**Authors:** Soumi De Montijo-Prieto, María del Carmen Razola-Díaz, Federica Barbieri, Giulia Tabanelli, Fausto Gardini, Maria Jiménez-Valera, Alfonso Ruiz-Bravo, Vito Verardo, Ana Mª Gómez-Caravaca

**Affiliations:** 1 Campus of Cartuja, Department of Microbiology, University of Granada, 18071 Granada, Spain; 2 Campus of Cartuja, Department of Nutrition and Food Science, University of Granada, 18071 Granada, Spain; 3 Biomedical Research Center, Institute of Nutrition and Food Technology ‘José Mataix’, University of Granada, Avda del Conocimiento sn, 18100 Granada, Spain; 4 Department of Agricultural and Food Sciences, University of Bologna, 40127 Bologna, Italy; 5 Interdepartmental Centre for Industrial Agri-Food Research, University of Bologna, 47521 Cesena, Italy; 6 Department of Analytical Chemistry, Faculty of Sciences, University of Granada, Avd. Fuentenueva s/n, 18071 Granada, Spain

**Keywords:** lactic acid fermentation, avocado agro waste, phenolic compounds, antioxidant activity, submerged fermentation

## Abstract

The growing global consumption of avocados, associated with contents including bioactive compounds with numerous health-promoting properties, is producing a large amount of agro wastes around the world. Different management approaches are available for the recovery of bioactive compounds from wastes as potential ingredients for use in the production of functional foods and nutraceuticals. Lactic acid fermentation can be used to exploit nutritional potential and add value to agro wastes. In this study, fermentations with lactic acid bacteria were carried out in avocado leaves, and the total phenolic content and the antioxidant activity were determined by DPPH and FRAP assays from hydroalcoholic extracts obtained from fermented avocado leaves. Fifteen new phenolic compounds were identified for the first time in avocado leaves by HPLC-ESI-TOF-MS. *L. plantarum* CECT 748T and *P. pentosaceus* CECT 4695T showed the highest antioxidant activity. The sum of phenolic compounds was increased by 71, 62, 55 and 21% in fermentations with *P. pentosaceus* CECT 4695T, *L. brevis* CECT 5354, *P. acidilactici* CECT 5765T and *L. plantarum* CECT 9567, respectively, while it was reduced in the fermentation with *L. plantarum* 748T by 21% as demonstrated by HPLC-ESI-TOF-MS. Biotransformations induced by bacterial metabolism modified the phenolic compound profile of avocado leaves in a strain-specific-dependent manner. *P. pentosaceus* CECT 4695T significantly increased kaempferol, *P. pentosaceus* 4695T, *L. brevis* 5354 and *L. plantarum* 9567 increased rutin, and dihydro-*p*-coumaric acid was increased by the five selected lactic acid bacteria. Total flavonoids were highly increased after fermentations with the five selected lactic acid bacteria but flavonoid glucosides were decreased by *L. plantarum* 748T, which was related to its higher antioxidant activity. Our results suggest that lactic acid bacteria led the hydrolysis of compounds by enzymatic activity such as glycosidases or decarboxylase and the release of phenolics bound to the plant cell wall, thus improving their bioavailability.

## 1. Introduction

Avocado (*Persea americana* Mill., *Lauraceae*) is native to southern Mexico, but it is cultivated around the world including in Central and South America, Indonesia, the United States, Australia, South Africa and Spain [1,2], with the Hass variety the most cultivated in the world [3]. In 2021, the Food and Agriculture Organization of the United Nations (FAO) calculated that global avocado production was 8.69 million metric tonnes, overcoming the previous year’s production of 8.06 million metric tonnes (https://www.fao.org; accessed on 29 December 2022). Due to the increase in the avocado fruit’s production and consumption, the amount of agro waste is increasingly becoming a major problem for the environment [1,4]. During harvesting, avocado leaves are discarded without any industry application [5]; however, the extracts obtained from avocado leaves contain appreciable amounts of bioactive compounds such as alkaloids, triterpenoids, saponins, carbohydrates, fatty acids and polyphenols [6,7,8,9,10]. Plant phenolic compounds are found in high concentrations in plant leaves and the green stems, with them being affected by genetic factors, environmental conditions, geographic location or physiological variations [11]. Redox properties of phenolic compounds are related with their ability to neutralize free radicals, contributing to the amelioration of some degenerative diseases associated to oxidative stress, such as neurodegenerative and cardiovascular diseases, cancer or skin aging through photoprotection from UV rays [12]. They have been also associated with antibacterial and antifungal activities. Hydroalcoholic leaf extracts of seven Mexican cultivars of *P. americana* var. *drymifolia* showed strong antioxidant activity by DPPH and ABTS assays [13], and purified phenolic fractions of avocado leaf extract showed a concentration-dependent antibacterial effect [5]. Plant phenolics are found covalently bonded to plant cell walls and their functional activities depend on their chemical structure and bioavailability. In the human gut, dietary phenolics are transformed by fermentative microbiota, which plays an important role in their absorption and in the modulation of their functional properties with benefits to health [14,15].

Lactic acid bacteria (LAB) fermentation is used in the food industry for the enhancement of the nutritional quality of foods by increasing protein digestibility, mineral availability and the release of peptides and amino acids [16,17]. However, fermentation with selected lactic acid bacteria also increases the antimicrobial and antioxidant activities of foods [18,19]. LAB are part of the microbiota of raw vegetables and can be isolated from spontaneous vegetable fermentations [20]. Therefore, they are characterized by a good adaptation to different environments by the utilization of different substances [21,22] including plant niches [23]. The capability of LAB to metabolize plant material is species- and strain-specific [24], and it is affected by several factors such as the presence of fermentable substrates or the presence of inhibitory factors such as phenol compounds [23]. In fact, the different phenolic compounds and their concentrations can affect the metabolism and viability of LAB [25]; therefore, tolerance to high levels of phenols is required to utilise and to biotransform plant material [23]. The ability of the microorganisms to produce enzymes such as glucosidase, amylase, cellulase, tannase, chitinase or lipase during fermentations can break down/hydrolyse the bound compounds that are released during the extraction [14]. Moreover, the metabolization of phenolics by LAB is recognised as a stress tolerance mechanism for the detoxification and bioconversion of polyphenols [26,27].

For this reason, this study aimed to evaluate the effect of submerged fermentation with LAB on the phenolic profile of avocado leaves as a strategy to obtain phenolic-compound-enriched extracts with improved antioxidant properties.

## 2. Materials and Methods

### 2.1. Chemicals and Samples

HPLC (High-Performance Liquid Chromatography)-grade water and other reagents and solvents were purchased from Merck KGaA (Darmstadt, Germany). Water was purified using a Milli-Q system (Millipore, Bedford, MA, USA). All the analytical standards were purchased from Sigma-Aldrich (St. Louis, MO, USA).

Avocado leaf samples of variety Hass were collected from Salobreña (Spain, 36°44′48″ N 3°35′13″ W) in April 2022. The fresh leaves were air dried at room temperature in a dark environment and milled and sieved to 100 µm particle size; after that, they were frozen at −18 °C until the analyses.

### 2.2. Lactic Acid Bacteria Strains and Culture Media

Lactic acid bacteria (LAB) strains used in fermentations were obtained from the Spanish Collection of Type Cultures (CECT): *Pediococcus acidilactici* 5765T, *Pediococcus acidilactici* 98, *Pediococcus pentosaceus* 4695T, *Pediococcus pentosaceus* 923, *Leuconostoc mesenteroides* subsp. *mesenteroides* 219T, *Leuconostoc mesenteroides* subsp. *mesenteroides* 215, *Levilactobacillus brevis* 4121T, *Levilactobacillus brevis* 5354, *Lactiplantibacillus plantarum* subsp. *plantarum* 748T and *Lactiplantibacillus plantarum* 9567 (formerly strain C4). Strains were reactivated by incubation in MRS broth and agar, at 26 °C for 24–48 h, and stocks were maintained in glycerol at −20 °C.

### 2.3. Preparation of the Inocula

Strain stocks were cultivated in MRS broth and incubated at 26 °C for 24 h. After growth, the cells were harvested by centrifugation (3000× *g* for 20 min) to remove the medium and resuspended in 10 mL of sterile saline solution (0.85% NaCl). The bacterial concentration was estimated by turbidimetry and the suspensions were used as inocula for each fermentation. An aliquot was taken from each suspension for counting viable bacteria by enumeration of colonies on MRS agar plates.

### 2.4. Fermentation of Avocado Leaves

Fermentations were carried out as follows: 1 g of the dried ground avocado leaves were submerged in 8 mL of sterile water previously heated to 90 °C. After mixing and cooling, the mixture was supplemented with 1 mL sterile medium containing glucose and yeast extract to obtain a concentration of 0.4% *w*/*v* of each. Inocula were added to obtain a concentration between 10^6^ and 10^7^ cell/mL. CFU/mL counting on MRS agar and pH values were determined at 0, 24, 48, 72 and 96 h of incubation at 26 °C. A control without the addition of LAB strains was included. Two replicates were prepared for each bacteria and control. At the end of incubations, samples and control were stored at −20 °C and then freeze-dried for further determinations.

### 2.5. Polar Compound Extraction

Briefly, 0.2 g of lyophilized avocado leaf fermented powder was dissolved in a 6 mL solution of ethanol/water 80/20, *v*/*v*. The mixture was placed in an ultrasonic bath for 15 min, and then it was centrifuged for 10 min at 9000 rpm. The extracting procedure was repeated twice more and all the supernatants were collected, evaporated and reconstituted in 1 mL of methanol/water (50:50, *v*/*v*). The final extracts were filtered with regenerated cellulose filters 0.2 µm (Millipore, Bedford, MA, USA) and stored at −18 °C until the analyses.

### 2.6. Determination of Folin–Ciocalteu Reacting Substances

Folin–Ciocalteu spectrophotometric method was used to determine the total Folin–Ciocalteu reacting substances (FCRS) for the first screening in all the fermented avocado leaves [28]. Briefly, 500 µL of the Folin–Ciocalteu reagent was added to 100 µL of the extract. It was added to 6 mL of bi-distilled water and the flask was agitated for a minute. After that, it was added to 2 mL of 15% (*w*/*v*) Na_2_CO_3_ and filled up to 10 mL with bi-distilled water. The flasks were kept in darkness for 2 h and the measures were carried out at 750 nm and 25 °C with a UV–visible spectrophotometer (Spectrophotometer 300 Array, UV–Vis, single beam, Shimadzu, Duisburg, Germany). Calibration curve was carried out with gallic acid from 1 to 1000 ppm and the equation obtained was y = 0.0012x − 0.0164 (R² = 0.9984). Analyses were performed in triplicate and the results are expressed as mg gallic acid equivalents (GAE)/g dry weight (d.w.).

### 2.7. Determination of Antioxidant Activity: DPPH and FRAP Assays

DPPH and FRAP assays were carried out to determine the antioxidant capacity of the avocado leaf fermented by different strains’ extracts by the procedures described in previous research [29,30]. Briefly, for the DPPH antioxidant assay, it was mixed with 100 µL of extract with 2.9 mL of the DPPH reagent and the decrease in absorbance was measured after 30 min at 517 nm. Otherwise, for FRAP it was mixed with 30 µL of extract with 90 µL of distilled water and 900 µL of FRAP reagent, kept at 37 °C for 30 min and the absorbance was measured at 595 nm. The measurements were performed using a UV–visible spectrophotometer (Spectrophotometer 300 Array, UV–Vis, single beam, Shimadzu, Duisburg, Germany). In both assays, Trolox was used as the standard for the calibration curves from 1 to 1000 ppm and the equations obtained were y = 0.0027x + 0.0495 (R² = 0.9989) and y = 0.0031x + 0.0016 (R² = 0.9934) for the DPPH and FRAP assays, respectively. The analyses were performed in triplicate and the results are expressed in mg of Trolox equivalents (TE)/g of dry weight (d.w.).

### 2.8. Determination of Polar Compounds by HPLC-ESI-TOF-MS

Phenolic compounds present in the fermented and non-fermented avocado leaf extracts were analysed using an Acquity Ultra Performance Liquid Chromatography (UPLC) system (Waters Corporation, Milford, MA, USA) coupled to an electrospray ionization (ESI) source operating in the negative mode and a mass detector time of flight (TOF) micro mass spectrometer (Waters). The compounds of interest were separated on an ACQUITY UPLC BEH Shield RP18 column (1.7 μm, 2.1 × 100 mm; Waters Corporation, Milford, MA, USA) at 40 °C using the conditions and gradient previously stated [31]. H_2_O acidified with 1% of acetic acid and acetonitrile were used as phase A and B, respectively. Analyses were performed in triplicate. The identification of the phenolic compounds was made according to the literature. For ensuring the mass accuracy, the tolerances chosen had a score higher than 90% and error lower than 5 ppm. To quantify the phenolic compounds identified in the avocado leaf extracts, calibration curves were used for vanillic acid (y = 8.1947x + 122.91; R² = 0.9976), chlorogenic acid (y = 85.138x + 135.16; R² = 0.9978), ferulic acid (y = 16.507x + 92.06; R² = 0.9980), quercetin (y = 112.8x + 287.12; R² = 0.9957), catechin (y = 41.108x + 335.6; R² = 0.9959) and rutin (y = 26.176x + 403.46; R² = 0.9924). The results are expressed as µg/g d.w.

### 2.9. Data Processing

The data for the identification of polar compounds in the avocado leaves by HPLC-ESI-TOF-MS were elaborated using MassLynx 4.1 software (Waters Corporation, Milford, MA, USA). Statistical differences (Tukey test) by one-way ANOVA analysis, and Pearson correlations were performed using Statistica 7.0 package (StatSoft, Tulsa, OK, USA). The rest of the statistical analyses were performed using MetaboAnalyst 5.0.

## 3. Results and Discussion

### 3.1. Screening of Lactic Acid Bacteria in Fermentation of Avocado Leaves

As described above, a minimal medium (dextrose plus yeast extract) was added to stimulate the initiation of growth of the inoculated LAB. To check if the treatment with hot water was able to eliminate the microorganisms present in the avocado leaves, viable microorganism counts were performed on MacConkey agar (selective medium for enterobacteria), Tryptic soy agar (TSA, enriched medium for bacteria), and Sabouraud agar (medium for fungi). After incubation of the media, the counting was under the limit of detection of the tests.

As shown in Table S1, avocado leaves did not support the growth of most of the inoculated LAB strains. The number of viable bacteria dropped during the first hours of incubation and continued to decrease gradually throughout the fermentation, with the exception of the two *L. plantarum* strains, which increased their viable counts. While *L. plantarum* 748T reached its growth peak at 24 h of incubation (8.44 ± 0.01 log CFU/mL), the exponential phase of growth of *L. plantarum* 9567 was prolonged until 48 h reaching similar counts (8.41 ± 0.05 log CFU/mL). The growth of the two strains of *L. plantarum* caused the acidification of the medium (Figure S1). After 24 h of incubation, the pH dropped from 5.94 to 5.31 with *L. plantarum* 9567 and to 3.93 with *L. plantarum* 748T. After 48 h, both pH values were around 4. The pH values for the rest of the strains remained similar to the initial values. Although *P. acidilactici* strains did not increase their concentrations, they were kept viable during fermentations similar to the initial concentrations.

Poor bacterial growth in avocado leaves can be due to their composition. Some compounds present in fermentation, such as phenolics, can affect the viability and metabolism of LAB [23,32]. Avocado leaves contain glycosides, alkaloids, tannins, saponins, flavonoids, terpenoids and steroids [7,8,9,33] and represent a potential source of antibacterial molecules [5]. Nevertheless, high tolerance to phenolic compounds is found in LAB, especially in members of Lactobacilli [34], which can be isolated from fermented products with a high content of phenolic compounds [35]. *L. plantarum* has been widely studied for its adaptation to plant habits and capability to metabolise phenolics [24], and it is used as starter in food fermentation.

All the fermented avocado leaves at different hours of incubation were analysed in terms of Folin–Ciocalteu reacting substances (FCRS) and antioxidant activity, and the results are presented in Table 1.

As can be seen from the results, the FCRS content ranged from 17.34 to 30.72 mg GAE/g d.w. Otherwise, the antioxidant activity was in the range of 25.56–53.88 and 50.34–96.61 for DPPH and FRAP, respectively. In our study, we applied a heat treatment on the avocado leaves in order to eliminate contaminants that could affect the fermentation process. Thus, avocado leaves were submerged into hot water and then allowed to cool spontaneously. This treatment was carried out both on avocado leaves fermented with lactic bacteria and on the unfermented control. Yamassaki et al. previously reported no decrease in the phenolic content or antioxidant activity when heating avocado leaf hydroalcoholic solutions at 40–100 °C for more than 8 h, and the total phenolic content or antioxidant activity of the extracts did not decrease [36].

An increase in the FCRS content was detected after the fermentation for some microorganisms; however, antioxidant activity by DPPH and FRAP assays was lower after fermentations with LAB strains. For most strains, the highest antioxidant activity was found at 24 and 48 h of fermentation. According to the results, the highest antioxidant activity was obtained after 24 h fermentation with *P. acidilactici* 5765T in the DPPH assay with 50.01 ± 0.23 mg TE/g d.w. and after 48 h fermentation with *L. plantarum* 748T with 96.61 ± 1.60 mg TE/g d.w. in FRAP assay. It was previously reported that fermentation of avocado puree with *L. plantarum* resulted in high levels of total free amino acids and a marked increase in antioxidant activity [37]. However, studies of lactic acid fermentations of avocado leaves are scarce.

Among all the microorganisms tested, the two varieties of *L. mesenteroides* showed the most minor results at all the different hours evaluated, so they were discarded for the next steps. Regarding the two *L. brevis* strains, a significant reduction in the FCRS and the antioxidant activity for the 4121T strain was seen; conversely, the 5254 strain produced an increase in FCRS content. Comparing *P. acidilactici* 5765T and *P. acidilactici* 98, in the first case, a higher recovery of FCRS was found compared to the control; in the second case, a low amount of FCRS was noticed. When fermenting the avocado leaves with *P. pentosaceus* strains, the highest results were found at 24 h of fermentation with *P. pentosaceus* 4695T. Finally, *L. plantarum* 748T had the best FCRS at 48 h and *L. plantarum* 9567 at 24 h. Both strains of *L. plantarum* showed very good performances with significant differences to the control.

The FCRS content was related to the antioxidant activity. A significant positive correlation (*p* < 0.05) was found between total phenolic content and DPPH (r = 0.7857) and FRAP (r = 0.8069) assays. Likewise, the DPPH assay showed a significant positive correlation with the FRAP assay (r = 0.6107).

Based on the FCRS content and antioxidant capacity, the strains *P. acidilactici* 5765T (24 h), *P. pentosaceus* 4695T (24 h), *L. brevis* 5354 (24 h), *L. plantarum* 748T (48 h) and *L. plantarum* 9567 (24 h) were selected for studying the phenolic composition.

### 3.2. Identification of Polar Compounds in Fermented Avocado Leaves by HPLC-ESI-TOF-MS

**Figure 1:**
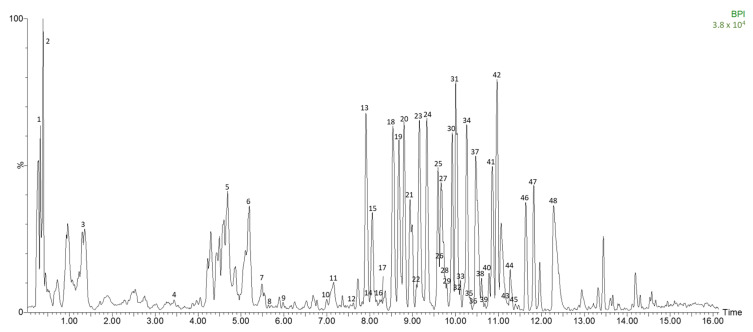
Base peak chromatogram of fermented avocado leaf by HPLC-ESI-TOF-MS.

The selected fermented avocado leaves and a control were characterized by HPLC-ESI-TOF-MS and a total of 48 polar compounds were identified. Among them were seven phenolic acids, thirty-seven flavonoids and four other compounds. They are presented in Table 2 with their experimental and calculated *m*/*z*, time (min), error (ppm), score (%), molecular formula and tentative name for each compound. The peaks presented in Table 2 correspond to the numbers shown in Figure 1, which is a representative chromatogram of a fermented avocado leaf. To the best of our knowledge, 15 polar compounds are identified here for the first time in avocado leaves.

Phenolic acids. Corresponding to peaks 3, 4, 5 and 7 were detected protocatechuic acid-4-glucoside, *p*-coumaric acid, chlorogenic acid and sinapic acid-C-hexoside in concordance with López-Cobo et al. [38] who previously identified them in avocado by-products. Moreover, at times 6.02 and 7.14, and with the *m*/*z* 221 and 165, two coumaric acid derivatives were found named as *p*-coumaroyl glycolic acid and dihydro-*p*-coumaric acid, respectively, according to the phenol explorer database [39]. The first one was previously quantified in lentils seeds [40] and the second one in olives [41], and both were found in avocado leaves here for the first time. In addition, a ferulic acid derivative tentatively named as dihydroferulic acid 4-O-glucuronide was detected with the *m*/*z* 371, the *m*/*z* in source fragment 195 and the predicted molecular formula C_16_H_20_O_10_ in agreement with Hu et al. [42] who found it in sweet cherries. Previously, Fan et al. [43] reported a similar compound named ferulic acid 4-O-glucoside in rejected avocados, but it is the first time this compound has been found in avocado leaves.

Flavonoids. Flavan-3-ols are a well-known group of flavonoids usually found in avocado samples, and in this case catechin derivatives were detected corresponding to peaks 8, 11 and 41, named as procyanidin dimer, procyanidin trimer and catechin diglucopyranoside, respectively [13]. Moreover, special attention was paid to the compounds detected at peaks 14 and 17. They were tentatively named as cinchonain-1a-(4beta->8)-catechin isomer a and b according to their *m*/*z* in source fragments 289 [C_15_H_13_O_6_]^−^ and 245 [M–3H]^3−^ (PubChem CID: 442686), and are described in avocado leaves here for the first time. Moreover, the flavonolignan cinchonain was identified at 8.15 min with the *m*/*z* 451 [13]. At time 7.57, the compound quercetin (*m*/*z* 301) was identified, and a total of 12 quercetin derivatives were detected. With the molecular formula C_27_H_30_O_17_, two isomers named as quercetin-diglucoside isomers a and b were found. Three isomers of quercetin 3-O-arabinosyl-glucoside (a, b and c) were detected with the *m*/*z* 595. Corresponding to peaks 23 and 24, two other isomers of a quercetin derivative were identified and named as quercetin-3-glucoside isomers a and b, respectively. In addition, quercetin glucuronide and quercetin-O-deoxyhesoxide were found at times 9.65 and 10.45. All of them were identified in agreement with Castro-López et al. [13] who previously described them in avocado leaves. Also they were found three other quercetin derivatives not reported previously in avocado samples. At 11.02 min the compound named as quercetin 3-O-acetyl-rhamnoside was identified according to Mi et al. [44] who found it in berries. With the *m*/*z* 565 and the molecular formula C_25_H_26_O_15_, the compound quercetin 3-xilosyl-(1->2)-alpha-L-arabinopyranoside, and with the *m*/*z* 505 and the formula C_23_H_22_O_13_, the compound quercetin 3-O-glucose-6″-acetate, were tentatively identified according to the PubChem database (PubChem CID: 44259231 and 24211981, respectively) with their *m*/*z* in source fragment 301. There were three isomers of the flavonoid rutin named as isomer a, b and c detected, corresponding to peaks 20, 21 and 31, respectively, with the *m*/*z* 609. Four isomers of a luteolin derivative were found with the molecular formula C_26_H_28_O_15_ that were called luteolin 7-O-(2″-O-pentosyl)-hexoside isomers a, b, c and d. At 11.81 min with the *m*/*z* 563, the compound apigenin-C-hexoside-C-pentoside was identified [13]. Otherwise, kaempferol was detected at 12.3 min with the molecular formula C_15_H_10_O_6_. Its derivatives, kaempferol-O-hexoside isomers a and b, kaempferol 3-O-acetyl-glucoside and kaempferol-O-coumaroyl, were identified corresponding to peaks 30, 34, 44 and 46, respectively [13]. With the *m*/*z* 461, the compound kaempferol 3-glucuronide that was previously quantified in strawberry [45] and endive [46] was tentatively identified. Additionally, two isomers (a and b) of kaempferol 3,4′-dixyloside were detected at 10.49 and 10.57 min, respectively, in agreement with Nakane et al. [47] who identified them in leaves of *Allium macrostemon*. In addition, the compounds detected at peaks 43 and 45 were tentatively named as luteolin 7-[6-O-(2-methylbutyryl)-beta-glucoside] isomers a and b, respectively, according to its fragment 285 that corresponds to C_15_H_10_O_6_ (luteolin), and the other to its source fragments 191 and 339 in agreement with Xiong et al. [48] who identified it in sorghum.

Other. Three organic acids and two quinones were detected. Corresponding to peaks 1 and 2, two isomers (a and b) of quinic acid, respectively, were identified [38,49]. Moreover, with the *m*/*z* 299, the compounds found at 9.62 and 10.06 min were tentatively identified as two isomers of a trihydroxyanthraquinones named as emodic acid according to its *m*/*z* in source fragments 255 [C_14_H_8_O_5_]^−^, 243 [C_13_H_8_O_5_]^−^ and 227 [C_13_H_8_O_4_]^−^, and the PubChem database (PubChem CID: 361510).

### 3.3. Quantification of Phenolic Compounds by HPLC-ESI-TOF-MS and Its Biotransformations during Fermentation in Avocado Leaves

The phenolic acids and flavonoids identified in the unfermented and selected fermented avocado leaves were quantified and the results are summarized in Table 3.

Fermentations of avocado leaves with the five selected LAB resulted in modifications in their phenolic profile (Figure S2). Total phenolic compounds’ content was increased by 71, 62, 55 and 21% in fermentations with *P. pentosaceus* 4695T, *L. brevis* 5354, *P. acidilactici* 5765T and *L. plantarum* 9567, respectively, while it was reduced in the fermentation with *L. plantarum* 748T by 21% in comparison to the unfermented control. Total phenolic acids were increased by 27, 40 and 43% in *P. pentosaceus* 4695T, *L. brevis* 5354 and *L. plantarum* 9567 fermentations, respectively, but decreased with *L. plantarum* 748T and *P. acidilactici* 5765T. With regard to total flavonoids, their content was highly increased by 91, 96 and 75% in *P. acidilactici* 5765T, *P. pentosaceus* 4695T and *L. brevis* 5354 fermentations, respectively.

LAB are able to degrade and biotransform food phenolic compounds by tannase, amylase, esterase, β-glucosidase, phenolic acid decarboxylase (PAD), reductase, or benzyl alcohol dehydrogenase enzymes [14,20]. Hydroxycinnamic acids such as caffeic, *p*-coumaric or ferulic acids can be reduced into dihydrocaffeic, phloretic or dihydroferulic acids, respectively, or decarboxylated into vinyl derivatives by a phenolic acid decarboxylase enzyme (PAD), and subsequently reduced into ethyl derivatives [50]. While *p*-coumaric acid was increased in fermentations with *P. acidilactici* 5765T, *P. pentosaceus* 4698T and *L. brevis* 5354, it was reduced after fermentation by *L. plantarum* 9567 and consumed by *L. plantarum* 748T (<LOQ). Trans-*p*-coumaric and *cis*-ferulic acids were decreased by *L. plantarum* in cowpeas (*Vigna sinensis* L.) depending on the isomeric form of the acids; however, spontaneous fermentation increased them [51]. Likewise, *L. brevis*, *L. plantarum* and *P. pentosaceus* were able to metabolize *p*-coumaric and ferulic acids through decarboxylation [52]. In addition, *p*-coumaric acid was degraded until *p*-vinyl-phenol and it reduced derivative dihydro-*p*-coumaric acid (phloretic acid) by *L. plantarum* in cherry juice exhibiting a strain-specific metabolism [24]. We found a significant increase in the concentration of dihydro-*p*-coumaric acid with the five selected strains, especially with *P. acidilactici* 5765T, *P. pentosaceus* 4695T and *L. brevis* 5354, but the decrease in *p*-coumaric acid in *L. plantarum* 748T fermentation did not correspond to a higher accumulation of this reduced metabolite compared to the last three bacteria.

Hydroxycinnamic acids are found glycosylated in plants (esterified), covalently attached to the cell wall and as a soluble form in cytoplasm. The breakdown of the ester linkages between polymers release the free phenolic acids [14,53]. Cinnamoyl ester hydrolases, also known as cinnamoyl esterase, catalyse the hydrolysis reaction of hydroxycinnamoyl esters releasing free acids that will be new substrates for phenolic acid decarboxylases PAD [53]. In our study, *p*-coumaroyl glycolic acid, an ester of *p*-coumaric acid, was decreased (<LOQ) in the fermentation with *L. plantarum* 748T. As we mentioned above, this microorganism also consumed *p*-coumaric acid, suggesting an initial hydrolysis of the ester by a cinnamoyl esterase followed by a decarboxylase activity. Chlorogenic acid, also known as 3-caffeoylquinic acid, is the ester of caffeic and quinic acids, and was significantly decreased in fermentations with all bacteria, especially with *P. acidilactici* 5765T. Conversely to our results, *L. plantarum* consumed caffeic, *p*-coumaric, ferulic, protocatechuic and *p*-hydroxybenzoic acids except for chlorogenic acid [26]. The ability to hydrolyse cinnamoyl esters of some *L. plantarum* strains was related to the presence of two esterases with differences in their substrate range: Lp_0796 that hydrolyses esters of caffeic, *p*-coumaric, ferulic and sinapic acids, while Est_1092 was able to hydrolyse both hydroxycinnamoyl and hydroxybenzoyl esters [54]. This cinnamoyl esterase activity is important in the de-esterification process of dietary fiber in human and ruminal digestion, improving the antioxidant, antiinflammatory and antimicrobial activity of complex dietary compounds [14].

Rutin isomers were increased by *P. pentosaceus* 4695T, *L. brevis* 5354 and *L. plantarum* 9567, while they were reduced by *P. acidilactici* 5765T (<LOQ). Nevertheless, in fermentation with *L. plantarum* 748T, rutin isomers a and b were reduced, but isomer c was increased significantly. Kaempferol, kaempferol-3-glucoside, quercetin and quercetin-3-glucoside were released from rutin after fermentation with *Aspergillus awamori* in Litchi pericarp [55]. Our results showed a significant increase in quercetin-3-glucoside isomers in all fermentations with the exception of *L. plantarum* 748T. Likewise, a significant increase in kaempferol was found only in the fermentation with *P. pentosaceus* 4695T, suggesting that this microorganism led to a biotransformation towards this compound. The hydrolysis of rutin to kaempferol-3-rutinoside or quercetin-3-glucoside is catalysed by α-rhamnosidases and further hydrolysed by β-glucosidases to free kaempferol or quercetin [32]. However, free quercetin was found under the limit of quantification (<LOQ) in both fermented and unfermented avocado leaves.

The enzymatic activity of the bacteria can break down vegetable cell walls and release bound phenolics, improving their bioavailability and facilitating their extraction [14,22]. Flavonoids were the main phenolic compound found in avocado leaves and were highly increased after fermentations with LAB. Flavonoids are found predominantly as glycosylated conjugates in plants, mostly as quercetin and kaempferol [56]. With the exception of *L. plantarum* 748T, the concentrations of flavonoid glucosides such as luteolin-7-O-(2″-O-pentosyl)-hexoside isomer a, quercetin-diglucoside and quercetin-3-O-arabinosyl-glucoside were increased compared to the unfermented control. Conversely, luteolin-7-O-(2″-O-pentosyl)-hexoside isomer d, quercetin-3-O-arabinosyl-glucoside isomer b and quercetin-3-glucoside isomer b, were reduced significantly by *L. plantarum* 748T. In soybeans’ and mung beans’ fermentation with *L. plantarum* 748T, glycosylated isoflavones were deglycosylated into their respective aglycones increasing their bioavailability [57]. Likewise, cultures of *L. plantarum* 748T transformed food aryl glycosides: phloridzin, esculin, daidzin and salicin into aglycones with the exception of quercetin glucoside, which remained glycosylated after incubation. The deglycosylation was associated with an increase in the antioxidant activity [58]. Our results showed a decrease in the concentration of quercetin glucoside isomer b but not in isomer a, suggesting a glycosyl hydrolase activity dependent on the isomeric form. In the gut, conjugated glucosides are hydrolysed by the intestinal microbiota to be absorbed into their corresponding aglycone, which show higher activity than their precursor glycosides [23]. β-glycosidase activity is widespread among LAB and have a significant positive impact on fermented products, improving their flavour and fragrance [59]. *L. plantarum* is commonly found in the human gastrointestinal tract and is used as a starter in the fermentation of dairy products, vegetables and meats [20]. Glycosidase activity of *L. plantarum* has been associated with an improvement in the bioaccessibility and bioavailability of food phenolic compounds as well as with an increase in their antioxidant activity [58]. Although *L. plantarum* 748T decreased the total phenolic content of avocado leaves, it showed a significant decrease in aryl-glucosides such as: quercetin-3-glucoside isomer b, protocatechuic acid 4-glucoside, quercetin-3-O-arabinosyl-glucoside isomer b, luteolin 7-O-(2″-O-pentosyl)-hexoside isomer d and kaempferol-O-hexoside isomer b, suggesting a higher glycosidase activity than the rest of the strains. Otherwise, *L. plantarum* 9567 increased total phenolic compounds by 21% but did not show such marked glucosidase activity as *L. plantarum* 748T. The higher glycoside deglycosylation shown by *L. plantarum* 748T, and to a lesser extent by *L. plantarum* 9567, may be related to the high antioxidant activities found in the DPPH and FRAP assays in comparison with the rest of the bacteria.

**Figure 2:**
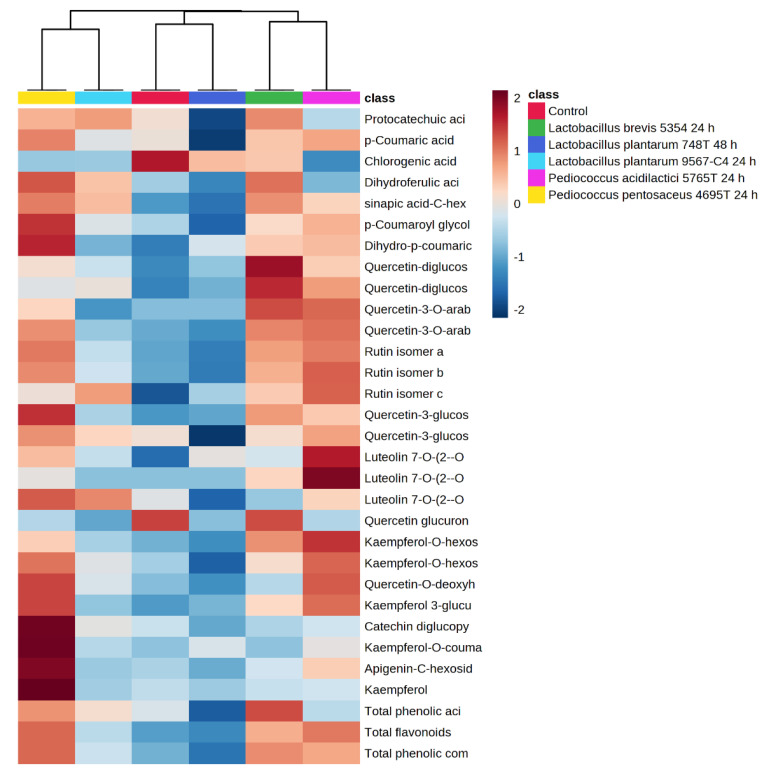
Clustering heatmap of the avocado leaves fermented by the selected strains and control.

A hierarchical clustering heatmap was performed to provide an intuitive visualization of all of the phenolic compounds quantified by HPLC-ESI-TOF-MS in the fermented avocado leaves by the selected strains and a non-fermented control. The features were previously normalized, the distance measure was the Pearson statistical meaning and the clustering method was the average. Therefore, the clustering result for the features (rows) and samples (columns) is shown in Figure 2. Each colour cell on the map corresponds to a concentration value normalized from 2 (intense red) to −2 (intense blue). Moreover, each sample has an associated colour (legend).

As can be seen from the figure, the avocado leaf fermented by *L. plantarum* 748T at 48 h was clustered with the control sample, which shows the minor differences among them in the polyphenol profile, and it had the lowest total phenolic content but with the highest content of chlorogenic acid. Close to them, *L. brevis* 5354 and *P. acidilactici* 5765T were also clustered according to their phenolic profile, being the group that showed higher contents of luteolin and quercetin derivatives. Finally, the group that was clustered furthest from the control was the one composed by *P. pentosaceus* 4695T and *L. plantarum* 9567. It seems to be a heterogeneous group in terms of amounts but with a similar profile and proportions between the individual phenolic compounds. Among them, *P. pentosaceus* 4695T was the strain that led the avocado leaf to release the highest content of coumaric acid derivatives and kaempferol derivatives with the highest total phenolic content. This clustering analysis confirms the strain-specific metabolism of LAB on the phenolic compounds present in avocado leaves, which is dependent on the capability of strains to tolerate and hydrolyse them.

## 4. Conclusions

This study allowed us to identify the chemical biotransformations induced by LAB strains in avocado leaves using submerged fermentations. A total of 48 polar compounds were identified by HPLC-ESI-TOF-MS and, to our knowledge, 15 of them were identified for the first time. We found a strain-specific metabolism of the phenolic compounds of avocado leaves, which was dependent on the tolerance of LAB strains to the phenolics’ concentration and their capacity to hydrolyse them. Fermentations with *P. acidilactici* CECT 5765T, *P. pentosaceus* CECT 4695T, *L. plantarum* CECT 9567 and *L. brevis* CECT 5354 led to an increase in the total phenolic content, with the exception of *L. plantarum* CECT 748T, which decreased it. The phenolic content in fermented leaf extracts was from 21 (*L. plantarum* CECT 9567) to 71% (*P. pentosaceus* CECT 4695T), higher than in the control. Briefly, submerged fermentation with lactic acid bacteria can be used in the exploitation and valorisation of avocado agro wastes for the production of enriched phenolic extracts.

## Figures and Tables

**Table 1:** Folin–Ciocalteu reacting substances’ content and antioxidant activity of all the strains tested from 24–96 h in avocado leaves and a control. Results are expressed as average ± standard deviation.

Microorganism	Time (min)	FCRS (mg GAE/g d.w.)	Antioxidant Activity (mg TE/g d.w.)	
			DPPH	FRAP
*L. mesenteroides* 215	24	17.34 ± 0.30 ^h^	27.84 ± 0.82 ^a,b^	57.86 ± 0.65 ^c,d^
	48	18.94 ± 0.13 ^g^	30.30 ± 0.06 ^b–e^	58.40 ± 0.97 ^d–f^
	72	20.23 ± 0.42 ^f^	33.34 ± 1.25 ^g–i^	72.96 ± 1.23 ^j^
	96	18.84 ± 0.33 ^g^	32.18 ± 1.27 ^e–g^	70.95 ± 1.21 ^j^
*L. brevis* 4121T	24	20.06 ± 0.16 ^f^	35.68 ± 0.52 ^i,j^	62.31 ± 1.04 ^e–h^
	48	19.85 ± 0.18 ^g^	33.17 ± 0.01 ^f– h^	58.29 ± 0.99 ^d,e^
	72	17.83 ± 0.19 ^h^	30.32 ± 0.70 ^c–e^	58.53 ± 0.99 ^d–f^
	96	19.31 ± 0.18 ^g^	29.23 ± 0.33 ^b–d^	60.57 ± 1.02 ^d–g^
*L. brevis* 5354	24	29.39 ± 0.60 ^a^	47.20 ± 1.54 ^r,s^	91.58 ± 1.53 ^p,q^
	48	27.62 ± 0.50 ^b^	43.78 ± 1.27 ^o–q^	86.34 ± 1.49 ^m–o^
	72	25.67 ± 0.42 ^c,d^	40.29 ± 1.36 ^l,m^	81.48 ± 1.39 ^k,l^
	96	19.87 ± 0.24 ^g^	31.64 ± 0.89 ^d–g^	63.43 ± 1.08 ^g,h^
*L. plantarum* 748T	24	21.98 ± 0.12 ^e^	25.56 ± 0.46 ^a^	71.58 ± 1.24 ^j^
	48	30.72 ± 0.52 ^a^	44.53 ± 1.00 ^p,q^	96.61 ± 1.60 ^r^
	72	30.07 ± 0.52 ^a^	43.35 ± 0.92 ^o–q^	92.01 ± 1.55 ^p,q^
	96	26.62 ± 0.40 ^c^	38.59 ± 1.04 ^k,l^	85.14 ± 1.45 ^l–n^
*L. plantarum* 9567	24	29.09 ± 0.02 ^a^	49.68 ± 0.78 ^t–v^	90.38 ± 1.57 ^o,p,q^
	48	27.65 ± 0.10 ^b,c^	42.74 ± 0.39 ^m,n^	93.23 ± 1.54 ^q,r^
	72	28.50 ± 0.30 ^b^	43.59 ± 0.66 ^o–q^	89.72 ± 1.51 ^o–q^
	96	27.08 ± 0.32 ^c^	40.82 ± 0.75 ^l–n^	89.10 ± 1.50 ^n–p^
*P. acidilactici* 5765T	24	29.56 ± 0.37 ^a^	51.32 ± 0.37 ^v^	78.50 ± 1.35 ^k^
	48	25.87 ± 0.08 ^c,d^	50.01 ± 0.23 ^u,v^	70.31 ± 1.19 ^j^
	72	24.49 ± 0.09 ^d^	42.85 ± 0.27 ^n–p^	50.34 ± 0.84 ^a^
	96	22.80 ± 0.05 ^e^	45.45 ± 0.37 ^q,r^	71.12 ± 1.20 ^j^
*P. acidilactici* 98	24	17.77 ± 0.01 ^h^	28.90 ± 0.07 ^b,c^	53.59 ± 0.90 ^a,b^
	48	20.10 ± 0.04 ^f^	33.09 ± 0.07 ^f–h^	56.66 ± 0.96 ^b–d^
	72	18.35 ± 0.53 ^g^	32.11 ± 1.28 ^e–g^	72.28 ± 1.21 ^j^
	96	18.58 ± 0.21 ^g^	31.33 ± 0.36 ^c–g^	64.51 ± 1.08 ^g,h^
*P. pentosaceus* 4695T	24	27.70 ± 0.20 ^b,c^	50.04 ± 0.56 ^u,v^	93.33 ± 1.57 ^q,r^
	48	21.49 ± 0.24 ^e^	48.03 ± 0.55 ^s–u^	82.79 ± 1.41 ^l,m^
	72	21.22 ± 0.16 ^e^	36.45 ± 0.41 ^j,k^	65.64 ± 1.11 ^h,i^
	96	17.46 ± 0.13 ^h^	36.63 ± 0.94 ^j,k^	53.82 ± 0.93 ^a–c^
*P. pentosaceus* 923	24	20.21 ± 0.02 ^f^	42.56 ± 0.74 ^m–p^	62.30 ± 1.03 ^e–h^
	48	21.60 ± 0.08 ^e^	41.38 ± 0.28 ^m–o^	68.89 ± 1.14 ^i,j^
	72	23.41 ± 0.21 ^d,e^	48.79 ± 0.28 ^s–u^	78.61 ± 1.35 ^k^
	96	23.53 ± 0.59 ^d^	49.70 ± 0.27 ^t–v^	83.41 ± 1.41 ^l,m^
*L. mesenteroides* 219T	24	20.49 ± 0.43 ^f^	47.47 ± 0.32 ^r–t^	72.51 ± 1.20 ^j^
	48	18.94 ± 0.25 ^g^	43.39 ± 1.09 ^o–q^	64.97 ± 1.08 ^h,i^
	72	20.08 ± 0.01 ^f^	35.38 ± 0.29 ^h–j^	62.42 ± 1.04 ^f–h^
	96	18.88 ± 0.11 ^g^	30.79 ± 0.50 ^c–f^	57.49 ± 1.00 ^b–d^
Control	-	26.90 ± 0.04 ^b,c^	53.88 ± 0.58 ^w^	92.10 ± 1.26 ^p,q^

Different letters in the same column mean statistically different (*p* < 0.05) values.

**Table 2:** Identified compounds in fermented and non-fermented avocado leaf by HPLC-ESI-TOF-MS.

Peak	Time (min)	*m*/*z* Experimental	*m*/*z* Calculated	Error (ppm)	Score (%)	Molecular Formula	Compound
1	0.324	191.0554	191.0556	−1.0	100	C_7_H_12_O_6_	Quinic acid isomer a
2	0.394	191.0549	191.0556	−3.7	100	C_7_H_12_O_6_	Quinic acid isomer b
3	1.362	315.0703	315.0716	−4.1	99.97	C_13_H_16_O_9_	Protocatechuic acid-4-glucoside
4	3.406	163.0398	163.0395	1.8	100	C_9_H_8_O_3_	*p*-coumaric acid
5	4.688	353.0865	353.0873	−2.3	99.97	C_16_H_18_O_9_	Chlorogenic acid
6	5.172	371.0977	371.0978	−0.3	95.46	C_16_H_20_O_10_	Dihydroferulic acid 4-O-glucuronide
7	5.478	385.1153	385.1135	4.7	90.68	C_17_H_22_O_10_	sinapic acid-C-hexoside
8	5.644	577.1351	577.1346	0.9	99.3	C_30_H_26_O_12_	Procyanidin dimer
9	6.016	221.044	221.045	−4.5	100	C_11_H_10_O_5_	*p*-Coumaroyl glycolic acid
10	7.138	165.0547	165.0552	−3.0	100	C_9_H_10_O_3_	Dihydro-*p*-coumaric acid
11	7.199	865.1981	865.198	0.1	91.8	C_45_H_38_O_18_	Procyanidin trimer
12	7.568	301.0342	301.0348	−2.0	96.02	C_15_H_10_O_7_	Quercetin
13	7.903	625.1406	625.1405	0.2	98.96	C_27_H_30_O_17_	Quercetin-diglucoside isomer a
14	7.973	739.1682	739.1663	2.6	90.78	C_39_H_32_O_15_	Cinchonain-1a-(4beta->8)-catechin isomer a
15	8.048	625.1428	625.1405	3.7	99.53	C_27_H_30_O_17_	Quercetin-diglucoside isomer b
16	8.147	451.1015	451.1029	−3.1	99.51	C_24_H_20_O_9_	Cinchonain
17	8.263	739.1646	739.1663	−2.3	95.25	C_39_H_32_O_15_	Cinchonain-1a-(4beta->8)-catechin isomer b
18	8.531	595.1297	595.1299	−0.3	94.55	C_26_H_28_O_16_	Quercetin-3-O-arabinosyl-glucoside isomer a
19	8.668	595.1292	595.1299	−1.17	95.67	C_26_H_28_O_16_	Quercetin-3-O-arabinosyl-glucoside isomer b
20	8.788	609.146	609.1456	0.7	99.01	C_27_H_30_O_16_	Rutin isomer a
21	8.966	609.1456	609.1456	0.0	96.23	C_27_H_30_O_16_	Rutin isomer b
22	9.07	595.1286	595.1299	−2.2	99.57	C_26_H_28_O_16_	Quercetin-3-O-arabinosyl-glucoside isomer c
23	9.144	463.0862	463.0877	−3.2	94.2	C_21_H_20_O_12_	Quercetin-3-glucoside isomer a
24	9.314	463.0866	463.0877	−2.4	99.16	C_21_H_20_O_12_	Quercetin-3-glucoside isomer b
25	9.57	579.1331	579.135	−3.3	96.32	C_26_H_28_O_15_	Luteolin 7-O-(2″-O-pentosyl)-hexoside isomer a
26	9.624	299.0183	299.0192	−3.0	98.09	C_15_H_8_O_7_	Emodic acid isomer a
27	9.645	477.0653	477.0669	−3.4	99.22	C_21_H_18_O_13_	Quercetin glucuronide
28	9.715	579.135	579.135	0.0	91.62	C_26_H_28_O_15_	Luteolin 7-O-(2″-O-pentosyl)-hexoside isomer b
29	9.765	565.1204	565.1193	1.9	92.13	C_25_H_26_O_15_	Quercetin 3-xilosyl-(1->2)-alpha-L-arabinopyranoside
30	9.901	447.0918	447.0927	−2.0	94.92	C_21_H_20_O_11_	Kaempferol-O-hexoside isomer a
31	9.992	609.1456	609.1456	0.0	99.88	C_27_H_30_O_16_	Rutin isomer c
32	10.06	299.0192	299.0192	0.0	99.87	C_15_H_8_O_7_	Emodic acid isomer b
33	10.087	579.1343	579.135	−1.2	92.58	C_26_H_28_O_15_	Luteolin 7-O-(2″-O-pentosyl)-hexoside isomer c
34	10.244	447.0915	447.0927	−2.7	93.19	C_21_H_20_O_11_	Kaempferol-O-hexoside isomer b
35	10.311	505.096	505.0982	−4.4	96.8	C_23_H_22_O_13_	Quercetin 3-O-glucose-6″-acetate
36	10.451	447.0913	447.0927	−3.1	93.55	C_21_H_20_O_11_	Quercetin-O-deoxyhesoxide
37	10.493	461.0706	461.0779	−3.0	99.87	C_21_H_18_O_12_	Kaempferol 3-glucuronide
38	10.567	549.124	549.1244	−0.7	92.41	C_25_H_26_O_14_	Kaempferol 3,4′-dixyloside isomer b
39	10.77	549.1262	549.1244	3.3	93.06	C_25_H_26_O_14_	Kaempferol 3,4′-dixyloside isomer a
40	10.845	579.136	579.135	1.7	94.44	C_26_H_28_O_15_	Luteolin 7-O-(2″-O-pentosyl)-hexoside isomer d
41	10.944	593.1521	593.1506	2.5	99.34	C_27_H_30_O_15_	Catechin diglucopyranoside
42	11.022	489.1032	489.1033	−0.2	93.58	C_23_H_22_O_12_	Quercetin 3-O-acetyl-rhamnoside
43	11.266	531.1507	531.1503	0.8	99.48	C_26_H_28_O_12_	Luteolin 7-[6-O-(2-methylbutyryl)-beta-glucoside] isomer a
44	11.291	489.1024	489.1033	−1.8	98.79	C_23_H_22_O_12_	Kaempferol 3-O-acetyl-glucoside
45	11.374	531.15	531.1503	−0.6	94.94	C_26_H_28_O_12_	Luteolin 7-[6-O-(2-methylbutyryl)-beta-glucoside] isomer b
46	11.618	431.0989	431.0978	2.6	93.85	C_21_H_20_O_10_	Kaempferol-O-coumaroyl
47	11.808	563.1408	563.1401	1.2	94.47	C_26_H_28_O_14_	Apigenin-C-hexoside-C-pentoside
48	12.313	285.0394	285.0399	−1.8	90.15	C_15_H_10_O_6_	Kaempferol

**Table 3:** Phenolic compounds quantified by HPLC-ESI-TOF-MS in the fermented avocado leaves and a control. Results are expressed as average ± standard deviation.

	µg/g d.w.					
Compound	*P. acidilactici* CECT 5765T	*P. pentosaceus* CECT 4695T	*L. brevis* CECT 5354	*L. plantarum* CECT 748T	*L. plantarum* CECT 9567	Control
Protocatechuic acid-4-glucoside	235.18 ± 10.3 a	364.60 ± 7.40 c	401.68 ± 7.01 d	<LOQ	384.61 ± 7.15 d	301.93 ± 2.66 b
Chlorogenic acid	37.84 ± 1.25 a	93.16 ± 0.66 b	194.14 ± 3.75 c	201.66 ± 3.66 c	95.63 ± 2.39 b	310.69 ± 5.89 d
Dihydroferulic acid 4-O-glucuronide	486.77 ± 6.16 b	766.37 ± 16.91 d	741.21 ± 6.43 d	419.82 ± 14.40 a	657.75 ± 6.57 c	516.74 ± 13.19 b
Sinapic acid-C-hexoside	115.99 ± 2.61 c	132.64 ± 2.93 d	129.81 ± 4.43 d	71.78 ± 1.16 a	120.71 ± 2.40 c	81.34 ± 1.54 b
*p*-Coumaric acid	185.67 ± 5.25 d	200.73 ± 0.05 e	169.85 ± 2.25 c	<LOQ	140.29 ± 2.42 a	147.97 ± 1.55 b
*p*-Coumaroyl glycolic acid	42.16 ± 1.94 d	56.81 ± 3.41 e	35.88 ± 0.36 c	<LOQ	30.27 ± 0.74 b	24.05 ± 0.55 a
Dihydro-*p*-coumaric acid	191.22 ± 4.13 d	230.99 ± 2.98 e	186.30 ± 2.94 d	165.49 ± 0.42 c	140.21 ± 5.34 b	121.23 ± 1.94 a
Cinchonain	<LOQ	<LOQ	<LOQ	<LOQ	<LOQ	<LOQ
Cinchonain-1a-(4beta->8)-catechin isomer a	<LOQ	<LOQ	<LOQ	<LOQ	<LOQ	<LOQ
Cinchonain-1a-(4beta->8)-catechin isomer b	<LOQ	<LOQ	<LOQ	<LOQ	<LOQ	<LOQ
Procyanidin dimer	<LOQ	<LOQ	<LOQ	<LOQ	<LOQ	<LOQ
Procyanidin trimer	<LOQ	<LOQ	<LOQ	<LOQ	<LOQ	<LOQ
Catechin diglucopyranoside	174.76 ± 2.87 a,b	231.20 ± 14.79 c	168.59 ± 8.56 a,b	156.56 ± 2.79 a	179.05 ± 0.94 b	173.43 ± 2.46 a,b
Quercetin	<LOQ	<LOQ	<LOQ	<LOQ	<LOQ	<LOQ
Quercetin-diglucoside isomer a	396.74 ± 5.37 e	370.98 ± 11.08 d	558.21 ± 4.90 f	277.86 ± 9.81 b	324.16 ± 5.66 c	214.69 ± 4.12 a
Quercetin-diglucoside isomer b	217.72 ± 10.52 d	170.00 ± 3.00 c	262.31 ± 3.76 e	124.72 ± 7.42 b	176.52 ± 1.49 c	98.53 ± 6.75 a
Quercetin-3-O-arabinosyl-glucoside isomer a	498.00 ± 79.73 d	394.58 ± 8.20 c	519.29 ± 8.56 d	270.03 ± 14.03 b	223.42 ± 10.62 a	269.02 ± 0.79 b
Quercetin-3-O-arabinosyl-glucoside isomer b	302.52 ± 10.79 d	284.07 ± 8.30 d	291.30 ± 7.83 d	103.57 ± 8.68 a	152.27 ± 10.32 c	126.63 ± 2.70 b
Quercetin-3-O-arabinosyl-glucoside isomer c	<LOQ	<LOQ	<LOQ	<LOQ	<LOQ	<LOQ
Quercetin-3-glucoside isomer a	248.31 ± 10.50 c	317.37 ± 16.93 e	272.94 ± 2.03 d	161.98 ± 4.52 ab	191.52 ± 4.31	153.37 ± 1.88 a
Quercetin-3-glucoside isomer b	281.07 ± 16.50 c	291.79 ± 15.11 c	225.80 ± 2.41 b	15.13 ± 2.87 a	237.81 ± 0.13 b	221.66 ± 12.70 b
Quercetin glucuronide	244.54 ± 4.62 a	245.23 ± 9.88 a	310.17 ± 15.05 b	234.06 ± 6.78 a	225.18 ± 3.68 a	312.69 ± 11.19 b
Quercetin 3-apiosyl-(1->2)-alpha-L-arabinopyranoside	<LOQ	<LOQ	<LOQ	<LOQ	<LOQ	<LOQ
Quercetin-O-deoxyhesoxide	223.65 ± 8.65 e	233.55 ± 2.55 e	123.24 ± 1.33 c	75.82 ± 6.74 a	140.25 ± 3.27 d	102.90 ± 2.56 b
Quercetin 3-O-glucose-6″-acetate	<LOQ	<LOQ	<LOQ	<LOQ	<LOQ	<LOQ
Quercetin 3-O-acetyl-rhamnoside	<LOQ	<LOQ	<LOQ	<LOQ	<LOQ	<LOQ
Rutin isomer a	348.26 ± 1.27 e	350.88 ± 3.37 e	324.72 ± 5.94 d	117.66 ± 8.92 a	219.94 ± 9.95 c	155.12 ± 10.53 b
Rutin isomer b	281.89 ± 0.47 f	257.55 ± 5.62 e	232.75 ± 6.29 d	54.63 ± 5.25 a	157.58 ± 7.31 c	94.67 ± 0.40 b
Rutin isomer c	557.92 ± 1.75 e	473.91 ± 11.52 c	498.48 ± 11.19 c	426.23 ± 10.28 b	527.58 ± 3.41 d	332.33 ± 13.44 a
Luteolin 7-O-(2″-O-pentosyl)-hexoside isomer a	184.54 ± 11.24 d	124.92 ± 9.70 c	88.77 ± 1.71 b	97.85 ± 0.37 b	82.09 ± 2.83 b	17.24 ± 3.20 a
Luteolin 7-O-(2″-O-pentosyl)-hexoside isomer b	<LOQ	<LOQ	<LOQ	<LOQ	<LOQ	<LOQ
Luteolin 7-O-(2″-O-pentosyl)-hexoside isomer c	<LOQ	<LOQ	<LOQ	<LOQ	<LOQ	<LOQ
Luteolin 7-O-(2″-O-pentosyl)-hexoside isomer d	146.00 ± 45.85 c	181.60 ± 14.25 d	108.99 ± 3.12 b	69.94 ± 7.67 a	169.59 ± 1.49 d	130.81 ± 3.40 c
Luteolin 7-[6-O-(2-methylbutyryl)-beta-glucoside] isomer a	<LOQ	<LOQ	<LOQ	<LOQ	<LOQ	<LOQ
Luteolin 7-[6-O-(2-methylbutyryl)-beta-glucoside] isomer b	<LOQ	<LOQ	<LOQ	<LOQ	<LOQ	<LOQ
Kaempferol	32.04 ± 3.57 b	232.24 ± 25.93 c	26.53 ± 4.71 a,b	<LOQ	2.82 ± 0.78 a,b	23.49 ± 0.99 a,b
Kaempferol-O-hexoside isomer a	309.41 ± 8.65 f	219.59 ± 9.05 d	258.68 ± 2.99 e	94.00 ± 6.02 a	148.73 ± 0.92 c	120.91 ± 7.55 b
Kaempferol-O-hexoside isomer b	258.40 ± 7.65 c	250.72 ± 11.78 c	177.49 ± 2.89 b	<LOQ	156.92 ± 9.50 b	116.48 ± 6.57 a
Kaempferol 3-glucuronide	154.26 ± 4.36 e	171.12 ± 1.78 f	101.26 ± 0.92 d	31.31 ± 5.45 b	40.93 ± 2.45 c	15.95 ± 2.62 a
Kaempferol 3,4′-dixyloside isomer a	<LOQ	<LOQ	<LOQ	<LOQ	<LOQ	<LOQ
Kaempferol 3,4′-dixyloside isomer b	<LOQ	<LOQ	<LOQ	<LOQ	<LOQ	<LOQ
Kaempferol 3-O-acetyl-glucoside	<LOQ	<LOQ	<LOQ	<LOQ	<LOQ	<LOQ
Kaempferol-O-coumaroyl	38.94 ± 4.85 c	135.91 ± 3.08 d	8.34 ± 1.69 a	34.16 ± 5.92 c	21.29 ± 7.20	8.27 ± 1.39 ab
Apigenin-C-hexoside-C-pentoside	63.37 ± 8.24 c	137.46 ± 3.42 d	36.65 ± 4.60 b	<LOQ	17.34 ± 4.26 a	22.80 ± 1.98 a
Sum of phenolic acids	1445.14 ± 32.50 b	1976.73 ± 30.92 e	2185.23 ± 27.17 f	886.58 ± 19.65 a	1686.24 ± 27.02 d	1557.08 ± 27.32 c
Sum of flavonoids	4937.17 ± 113.27 c,d	5026.32 ± 190.43 d	4592.16 ± 105.17 c	2355.58 ± 113.52 a	3417.55 ± 90.51 b	2606.36 ± 97.24 a
Sum of phenolic compounds	6382.32 ± 145.78 d	7003.05 ± 221.35 e	6777.39 ± 132.34 d,e	3242.17 ± 133.17 a	5103.78 ± 117.53 c	4163.45 ± 124.56 b

Different letters in the same row (a–f) mean statistical differences (*p* < 0.05); LOQ: limit of quantification.

## Data Availability

Data are contained within the article and Supplementary Materials.

## Supplementary Materials

The following supporting information can be downloaded at: https://www.mdpi.com/article/10.3390/antiox12020298/s1, Figure S1: pH values of lactic acid bacteria cultures during fermentation of avocado leaves; Figure S1: Phenolic profile in fermented and unfermented avocado leaves by HPLC-ESI-TOF-MS; Table S1: Log CFU/mL of lactic acid bacteria in avocado leaves.
