# Supplementary material for: Impact of Lactic Acid Bacteria Fermentation on Phenolic Compounds and Antioxidant Activity of Avocado Leaf Extracts
Source: Antioxidants (Basel). 2023 Jan 28;12(2):298. doi: 10.3390/antiox12020298 (PMC9952674; doi:10.3390/antiox12020298)
Supplement: Supplementary file 1 [file antioxidants-12-00298-s001.zip › antioxidants-2167187-supplementary.pdf]

Supplementary tables and figures

Table S1. Log CFU/ml of lactic acid bacteria in avocado leaves

	Log CFU/ml ± SD				
	**0 h**	**24 h**	**48 h**	**72 h**	**96 h**
P. acidilactici CECT 5765T	7.93 ± 0.03	7.86 ± 0.01	7,68 ± 0.05	7.75 ± 0.06	7.20 ± 0.07
P. acidilactici CECT 98	7.81 ± 0.08	7.47 ± 0.04	7.31 ± 0.05	6.66 ± 0.04	6.36 ± 0.06
P. pentosaceus CECT 4695T	7.25 ± 0.05	6.50 ± 0.03	6,36 ± 0.06	5.08 ± 0.04	4.25 ± 0.05
P. pentosaceus CECT 923	7.72 ± 0.05	6.50 ± 0.03	6.35 ± 0.06	5.08 ± 0.04	4.25 ± 0.05
L. mesenteroides CECT 219T	7.23 ± 0.08	6.26 ± 0.06	5.37 ± 0.06	5.07 ± 0.04	5.64 ± 0.05
L. mesenteroides CECT 215	4.30 ± 0.04	2.81 ± 0.13	5.21 ± 0.04	2.77 ± 0.07	1.09 ± 0.09
L. brevis CECT 4121T	8.45 ± 0.08	6.72 ± 0.03	6.63 ± 0.02	6.09 ± 0.05	5.36 ± 0.05
L. brevis CECT 5354	6.38 ± 0.04	5.24 ± 0.05	5.48 ± 0.06	5.21 ± 0.08	5.13 ± 0.05
L. plantarum CECT 748T	7.94 ± 0.04	8.44 ± 0.01	8.41 ± 0.05	8,30 ± 0.18	8.15 ± 0.14
L. plantarum CECT 9567	7.98 ± 0.02	6.79 ± 0.00	8.45 ± 0.02	8.48 ± 0.10	8.15 ± 0.14

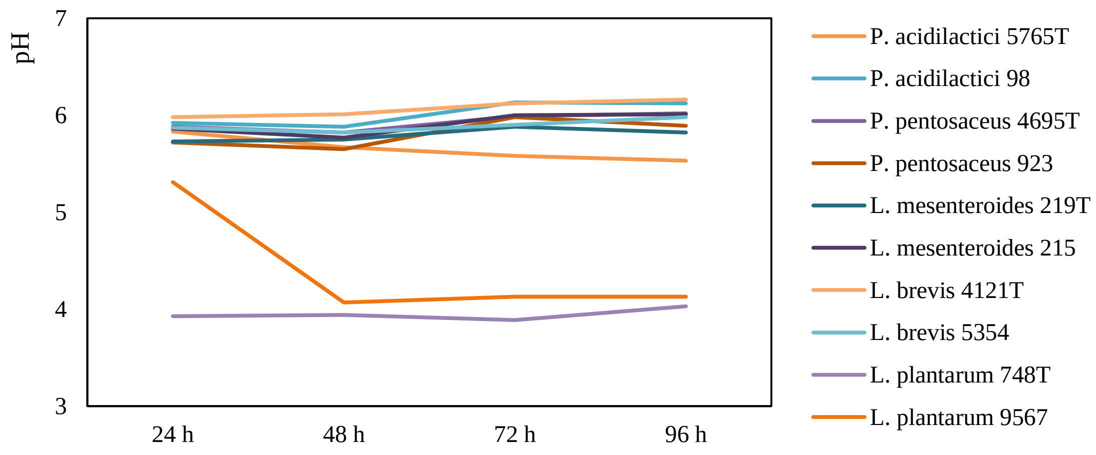

Figure S1. pH values of lactic acid bacteria cultures during fermentation of avocado leaves

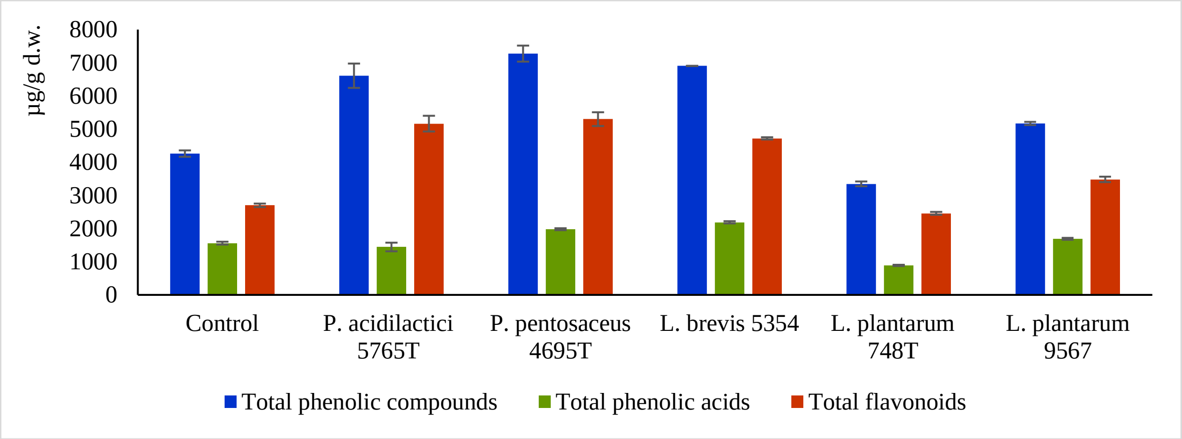

Figure S2. Phenolic profile in fermented and unfermented avocado leaves by HPLC-ESI-TOF-MS.
